# The Formation and Excretion of Uric Acid

**Published:** 1888-09

**Authors:** A. Haig

**Affiliations:** M.A. and M. D., Oxon., M.R.C.P., Physician to the Royal Hospital for Children and Women


### THE FORMATION AND EXCRETION OF URIC ACID, CONSIDERED WITH REFERENCE TO GOUT AND ALLIED DISEASES.

BY A. HAIG, M.A. AND M. D., OXON., M.R.C.P.,
Physician to the Royal Hospital for Children and Women.

Some researches on the excretion of uric acid, on which I have been engaged for several years, now seem likely to throw new light on the part played by this substance in the causation of disease.

The diseases to which my results more especially apply are gout, a certain form of headache (migraine) (*Med. Chi. Trans.*, vol. lxx), some cases of epilepsy (*Neurologisches Centralblatt*, March 1, 1888), and lastly, rheumatism, acute and chronic, though the evidence of uric acid causation is not equally strong in all.

My researches show (1) that the excretion of uric acid (*Journal of Physiology*, vol. viii, Nos. 3 and 4) can be controlled by drugs and foods in a way and to an extent not previously known ; that, whatever the amount excreted to-day, it may, within certain limits, be made either greater or less to-morrow.

2. That when uric acid excretion is diminished relatively to the urea, the diminution is due to retention in the liver and spleen, and possibly in some other organs and tissues, of part of the uric acid which was formed; and, conversely, when uric acid excretion is increased relatively to the urea, such increase is due to a washing out of a store previously accumulated in the above organs and tissues.

3. That the formation of uric acid bears an almost constant relation to that of urea, though its excretion can be varied by food and drugs to a considerable extent.

4. That uric acid is probably never in excess in the blood except when alkalies or other solvents wash it out in considerable quantities from the stores which have gradually accumulated in the above-mentioned organs.

5. That excess of uric acid in the blood, brought about in this manner, is a sufficient cause of many of the symptoms of the diseases I have mentioned, and probably of others also.

6. That drugs and foods which diminish the solubility of uric acid diminish its excretion and lead to retention and accumulation; and, conversely, foods and drugs which increase its solubility facilitate its excretion and prevent retention.

7. That drugs which diminish the solubility and excretion of uric acid are harmful in the diseases connected with it, while drugs which increase its solubility and excretion are useful in these same diseases.

I began my research by investigating a form of migraine, or, as I now prefer to call it, uric acid headache, from which I had been a sufferer all my life; and my first observation of importance was that leaving off butcher meat (*Practitioner*, Aug., 1884, and March, 1886) produced gradually-increasing immunity from attacks. This and the clinical relationship of the headache to gout led to my examining the urine and finding a very marked rise in uric acid excretion, corresponding to the headache (*Med.-Chir. Trans.*, vol. lxx).

I then began to speculate as to the cause of this uric acid fluctuation; and, having noticed the apparently great gout-producing power of ales, and their strong acid reaction, I was led to test the effects of acids, alkalies, and, finally, of many other drugs, on the excretion of uric acid, obtaining results some of which form the subject of this paper.

The processes used in my experiments are as follows: for urea, the hypobromate test, with the apparatus of Dupré, variations of temperature and pressure being allowed for; for uric acid, Professor Haycraft's process (see *Journal*, vol. ii, 1885, p. 1100); and for acidity, a solution of phenol-phthaleine with a graduated solution of soda.

I soon found that acids (*Journal of Physiology*, vol. viii, Nos. 3 and 4) always diminished the excretion of uric acid, and alkalies always increased it; and it thus became easy to see that butcher meat, beer, wine, etc., had made my headaches more frequent, because they caused a rise of acidity, prevented the free elimination of uric acid, and accumulated a store of it in the body, which on a future occasion would produce a headache. Alkalies, or a somewhat vegetarian diet, on the other hand, by facilitating the regular excretion of uric acid, would prevent any such troubles; and in proof of this, I found that by first accumulating uric acid with acids, and then washing it out into the blood with an alkali, I was able to produce the headache in question.

Sir A. Garrod (*Journal*, vol. i, 1883, p. 653 [Lumelian Lectures]) has discussed the gout-producing powers of wines and beers, and puts it down entirely to the unfermented matters they contain. In discussing the matter he says the light wines which contain a considerable quantity of acid vegetable salts do least harm, while the heavy wines, as those of Spain, do most harm and are almost devoid of vegetable salts.

Speaking of acidity, he says that fresh fruits diminish acidity, through conversion of their vegetable salts into carbonates, and this I think gives us the key to the whole matter.

Both the above kinds of wines are strongly acid, but in the light wines the acidity is due to vegetable salts which are converted in the body into carbonates and act as alkalies, so that they cause no retention of uric acid, and have, as observed, but little gout-producing power. The heavier wines, on the other hand, have an acidity due to more fixed acids ; they act as a dose of acid does, and have great gout-producing power; and the same applies to ales and stout, which are, as I have shown (*Med.-Chir. Trans.*, vol. lxx, p. 12 of paper), strongly acid and cause great retention of uric acid ; so that the acidity of these beverages explains their action in gout-production far better than their unfermented matters, the action of which is not very clear ; and in gouty patients who indulge in these beverages after an interval of care, the rise in acidity they occasion and the increase of arthritic pains can be watched.

Foods, again, influence acidity by the salts they contain (see Sir W. Roberts, *Urinary and Renal Diseases*, ed. iv, p. 56), fruits and vegetables, as we have seen, diminishing acidity, and animal foods containing large amounts of phosphates, sulphates, and chlorides increasing acidity. Some foods may also contain salts having a special solvent action on uric acid; for example, phosphate of soda.

Sir A. Garrod in the above-quoted lectures gives a very interesting table from Lehmann of the effects of different diets on the excretion of uric acid : thus

| | Urea. | Uric Acid. |
|---|---|---|
| Mixed diet | 32.4 | 1.1 |
| Animal diet | 53.1 | 1.4 |
| Vegetable diet | 22.4 | 1.0 |
| Non-nitrogenous diet | 15.4 | 0.7 |

and he draws the inference from it that uric acid is not so much influenced by the nature of the food as urea is; but if the uric acid excretion is regarded relatively to the urea this conclusion will, I think, have to be altered; and I now give the table with the addition of my figures, showing the relation of uric acid to urea on each diet :

| | Urea. | Uric Acid. | Relation. |
|---|---|---|---|
| Mixed diet | 32.4 | 1.1 | 1–29 |
| Animal diet | 53.1 | 1.4 | 1–38 |
| Vegetable diet | 22.4 | 1.0 | 1–22 |
| Non-nitrogenous diet | 15.4 | 0.7 | 1–22 |

A long series of observations on the relative excretion of uric acid to urea has convinced me that a relation of 1–33—that is, 1 grain of uric acid for 33 grains of urea, is about the average or normal; and taking a long period a result somewhat near this is obtained. Thus in four months I excreted 41,804 grains of urea and 1,281 grains of uric acid, relation 1–32.6; but daily variations in this period ranged as high as 1–19, or as low as 1–45; but over a long period these more or less balance each other.

To apply this to the above table, it will be seen that on mixed diet there was slightly excessive excretion of uric acid ; on animal diet considerably diminished excretion (retention), and on vegetable diet excessive excretion, some of the previous retention being washed out. And a continuance of the animal diet would obviously soon produce a store of uric acid sufficient to account for anything from a "bilious" to a gouty attack. The results in these tables are similar to those I have obtained with various diets, and the relation of uric acid to urea are just those I meet with in my everyday work.

I have been led to infer from all these facts, that while the formation of uric acid alters but little from its relation to urea of 1–33 (subject, I have no doubt, to some small variations), its excretion is subject to frequent and great fluctuations, which account for its action in the production of disease.

*Iodide of Potassium* has in five-grain doses but little action on the excretion of uric acid; in larger doses it acts apparently merely as an alkali. Iodine taken as a tincture has little or no direct effect.

*Phosphate of Sodium* is a well-known solvent of uric acid, and it does, just as one would expect, greatly increase its excretion; it is probably the chief solvent of uric acid which is naturally present in the body.

*Salicylates* have the very important action of rendering the excretion of uric acid independent of acidity—that is, a rise in acidity does not cause retention while salicylates are present in the blood; and this fact may explain much of the value of these salts in diseases connected with uric acid (see discussion on my paper in the *Proceedings of Roy. Med.-Chir. Soc.*, January to March, 1888, p. 325).

*Lead and Iron.*—The urates of these metals are very insoluble, and they cause, as one would expect, retention of uric acid, which, as we shall see, may explain their action in several diseases (paper read at Roy. Med. Chi. Soc., April 28, 1888, reported in *Journal*, April 28; and also Sir A. Garrod, *Journal*, vol. i, 1883, p. 495).

*Colchicum.*—This indirectly lowers acidity and facilitates the excretion of uric acid. It may also have a direct action on the nerves in relieving the pains of gouty arthritis.

Besides drugs and foods, there are other ways in which the acidity of the blood and tissue fluids may be raised and retention of uric acid brought about, as for instance the formation of acids which are known to occur in dyspepsia; but a more important source of acids is probably to be found in the skin, for a large amount of acid is daily eliminated in sensible and insensible perspiration, and Sir A. Garrod (*Gout and Rheumatic Gout*, ed. iii, p. 258) has shown that when this function of the skin is checked by cold the acidity of the urine at once rises, and *vice versa*, and I have often repeated this experiment, both intentionally and unintentionally, on myself. The application of these facts to the causation of gout by exposure to weather or other sources of chill is sufficiently obvious, and is precisely similar to the case of beer, wine, meat, etc., previously spoken of.

And now to apply some of these results to the explanation of the disease.

The causation of headache of which I have spoken is, I think, fairly well worked out; it can be produced at any time in those subject to it by causing a *plus* excretion of uric acid with alkalies, the *plus* excretion and headache being, as I believe, the signs of excess of uric acid in the blood. If there is no store of uric acid in the body, alkalies will fail to produce either the *plus* excretion or the headache. I have also found an identical uric-acid fluctuation in some cases of epilepsy (*Neurol. Cent.*, March 1, 1888), and I think it probable that uric acid may be the cause of the fits in these cases just as it is of the headache of which I have spoken. Sir A. Garrod (*Gout and Rheumatic Gout*, pp. 459–61) pointed out many years ago that he had found blood drawn during fits of epilepsy to contain excess of uric acid, and this observation affords important support to my belief that we may safely argue from excess of uric acid in the urine to its excess in the blood.

Diet and drug action also affords many important parallels between the headache of urid acid and epilepsy. Thus, the value of a vegetarian diet in epilepsy is amply attested, and I believe that not a few of those engaged in its treatment habitually order their patients to abstain from meat.

Iron is believed by many to do harm in epilepsy, and lead-poisoning sometimes causes epileptic fits. Iron, so far as I know, does harm in the uric acid headache; and both these metals cause, as we have seen, retention of the uric acid.

In a considerable number of cases the fits have been treated by diet and drugs intended to influence the uric acid factor, and so far with some apparent success; but as it is almost impossible to say when a fit has been caused and when it has been prevented, epilepsy presents a problem which is much more difficult to solve than that of the urid acid headache.

I have also been much struck by the large number of cases of epilepsy which have a strong family history of gout, rheumatism, or minor uric acid troubles.

Another result of my research is the conviction that a considerable amount of hypochondriasis, heaviness, depression, and irritability may be produced by causing a rush of uric acid into the blood and its excessive secretion, and that the same conditions may be cured or made to give place to the opposite feeling by driving the uric acid out of the blood by an acid, or something that causes retention. There is here, again, an important parallel between epilepsy (*Neurologisches Centralblatt*, March 1, 1888, p. 4) and the uric acid headache; for both are preceded by retention of uric acid, and, preceding both, certain bright and joyous feelings (*loc. cit.*) have been noticed, no doubt corresponding to this detention, just as they correspond to it when it is produced by acids.

And these mental effects of uric acid have led me to think that some forms (especially the paroxysmal ones) of melancholia and mania might yield some results to an investigation of their uric acid connections, though no doubt, in all such diseases, what may be at first functional and due to uric acid is at last associated with vascular and organic change which is incurable.

In this connection, it is very interesting to note that Dr. Broadbent, in his Croonian Lectures of 1887, says: "An unbroken series of gradations may be traced from the irritability and depression of spirits attending functional disorder of the liver up to complete melancholia with delusions"; and he further says this is accompanied by *plus* tension (of pulse), which, if not the cause, is at least an index of the condition of the system on which the mental state depends. These observations are of double interest to me—first, because of the unbroken series of gradations traced from conditions I can produce up to serious mental disorder; and, secondly, because they tend to confirm an opinion I have held for some time, that a high tension pulse is, to some extent and in the absence of other obvious causes, an index of excess of uric acid in the blood.

The relation of gout and uric acid is not a matter of doubt; but my researches give, I think, a better explanation of its intimate pathology than was previously obtainable by showing the way in which urates come to accumulate in the body, and that this accumulation is for the most part due to acids. We have also seen that acids may be introduced from without in food and drinks, may be formed internally in dyspepsia, or may be retained by deficient skin action, and that the result of the rise of acidity from any such cause is a retention of uric acid.

Sir A. Garrod has shown it to be probable (*Gout and Rheumatic Gout*, p. 292) that the cartilages and ligaments of joints are, not only to some extent extra-vascular, but are also less alkaline than the blood and tissue fluids; so that we can at once see why uric acid, which is brought to these parts in blood less than usually alkaline, should be liable to be thrown out of solution and retained in these parts, beginning generally in the smaller peripheral joints where the circulation is least active; and the same reasoning thus applies to retention of uric acid in the joints as was previously used in regard to its retention in the liver and spleen (*Journal of Physiology*, pp. 213, 214), and we now see how a dose of iron, causing retention, or a rise of acidity however produced, may precipitate an attack of gout. Also, why a uric acid headache, or an attack of melancholia or mania, may promptly clear up on the supervention of a gout attack; for the rise in acidity which drives uric acid out of the blood and cures either of the first two diseases drives it into the joint and produces the podagra.

Exercise rather tends to prevent gout, because it increases skin activity, which again reduces acidity and promotes elimination of uric acid; and the same skin activity, no doubt, explains why those who suffer from uric acid headache have less of it in summer than in winter. With rheumatism we stand on less firm ground, because uric acid has not been proved to be in excess in the blood in this disease; but I hope I shall be excused for believing, at least till careful investigation has proved the contrary, that with the help of the knowledge we now possess about the excretion of uric acid it may yet be found to be in excess in the blood in this disease.

The marked effect of salicylates in acute rheumatism seems to me a very strong point in favor of uric acid causation, and in favor of the disease being due to retention of uric acid by acids, the vicious circle being at once cut through by the salicylate, which permits its free excretion. There are, besides, important connections in the matter of family history, and in effects of drugs, meat, beer, weather, and exercise between gout and rheumatism which are not to be easily disposed of, except on the theory of a similar causation.

Why uric acid should produce in one patient gout, in another rheumatism, in a third headache, and in a fourth epilepsy, may depend to some extent on the time during which it is in excess in the blood, and the amount of such excess; but, beyond the uric acid, there are no doubt peculiarities in the structure and function of nerves or vessels in each individual which determine the point on which the poison shall act most forcibly, and so to some extent the result it produces; but, in all these diseases alike, such regulation of diet, exercise, clothing, and climate as shall prevent high acidity from bringing about continued retention and accumulation of uric acid will serve to keep off attacks, even though the other factors remain in operation.—*The British Medical Journal.*

